# Investigation of postoperative hypernasality after superiorly based posterior pharyngeal flap

**DOI:** 10.1186/s40902-018-0164-2

**Published:** 2018-08-30

**Authors:** Yu-Jeong Shin, Yongsoo Kim

**Affiliations:** 1 Research Institute of Speech Sciences, Chonbuk National University, 567 Baekje-daero, Deokjin-gu, Jeonju, 54896 South Korea; 2 Department of Oral and Maxillofacial Surgery, Institute of Oral Bioscience, School of Dentistry, Clinical Research Institute of Chonbuk National University Hospital, Chonbuk National University, 20 Gungiro Road, Duckjin-Gu, Jeonju, Chonbuk 54907 South Korea

**Keywords:** Nasalance, Nasometer, Cleft palate, Posterior pharyngeal flap, Pharyngoplasty

## Abstract

**Background:**

Velopharyngeal insufficiency that accompanies speech resonance and articulation disorders can be managed through several intervention methods such as speech-language therapy, prosthetic aids, and surgery. However, for patients with severe hypernasality, surgical interventions are highly recommended. Among available surgical techniques, the posterior pharyngeal flap is most common.

**Case presentation:**

Two adult males with high nasalance scores underwent superiorly based posterior pharyngeal flap surgery, followed by speech testing by an expert speech-language therapist. Nasalance scores and articulation accuracy were assessed up until 1 year after the surgery. Nasalance scores were measured five times using a nasometer, after which the average value was calculated.

**Conclusions:**

Consistent declines in hypernasality over time are not easy to explain since the pedicled pharyngeal flap narrowed over time, secondary to cicatrization. However, scar tethering of the soft palate in a posterior direction could reduce the velopharyngeal port size over time. Therefore, long-term follow-up with intensive speech therapy is suggested for patients with severe hypernasality.

## Background

Velopharyngeal insufficiency (VPI) involves inability to adequately close the velopharyngeal (VP) port, which consists of the soft palate, and posterior and lateral pharyngeal walls. Structural and functional abnormalities of the palate and pharyngeal wall may cause VPI which is commonly observed in individuals with cleft palate. Due multiple VPI-related problems such as feeding and swallowing difficulties, speech disorders, and chronic ear infections, most surgeons recommend reparative surgery before the age of 12~18 months for patients with cleft palates. However, in 5~20% of patients, VPI remains after primary repair [1, 2].

Speech disorders that frequently persist following repair surgery include articulation and resonance disorders. Speech resonance disorders involve abnormal coupling between the oral and nasal cavities due to a VPI or oro-nasal fistula; this coupling causes excessive nasal resonance during vowels and vocalic consonants. VPI also can affect articulation resulting in nasal rustle, weak pressure consonants, and compensatory misarticulations [3, 4].

The management of VPI-related speech disorders frequently involves speech-language therapy, prosthetics, surgical interventions, or a combination of several methods. Especially, in patients with severe hypernasality, surgical treatments can be considered in advance over other noninvasive methods [5, 6]. Multiple surgical techniques have been introduced, the most popular of which include re-push back palatoplasty, posterior pharyngeal wall augmentation, sphincter pharyngoplasty, and posterior pharyngeal flap. Many surgeons prefer the posterior pharyngeal flap because of its ability to form a bridge between the soft palate and posterior pharyngeal wall, effectively reducing the size of VP port. High rates of around 80–90% improvement in VP function have been reported [6–10]. However, hypernasality completely resolves in approximately 50% of patients who undergo pharyngeal flap surgery [11]. Furthermore, while speech intelligibility frequently improves over time, hypernasality may increase, even after surgery [12]. Long-term studies of posterior pharyngeal flap surgeries, with speech evaluation and outcomes, are lacking and more long-term studies are required.

Herein, we report on two adult patients who underwent superiorly based posterior pharyngeal flap surgery after cleft repair. We include outcomes obtained through long-term postoperative speech evaluations, which revealed consistent improvement of nasalance over time.

## Case presentation

Our cases are two adults with hypernasality, both of whom underwent cleft palate repair during infancy. After a comprehensive speech and language assessment, each subject underwent posterior pharyngeal flap surgery to correct an observed resonance disorder secondary to VPI.

The patient of case 1 was a 19-year-old male who had a primary repair for incomplete cleft palate at the age of 15 months. The patient received speech therapy for 6 months while he was a preschool period. The patient of case 2 was a 49-year-old male who underwent complete cleft lip and palate repair at age 3. At the time of presentation, he had two missing anterior maxillary teeth. Neither patient received regular speech-language therapy following his pharyngoplasty.

### Surgical procedure and cicatricial contraction

**Fig. 1:**
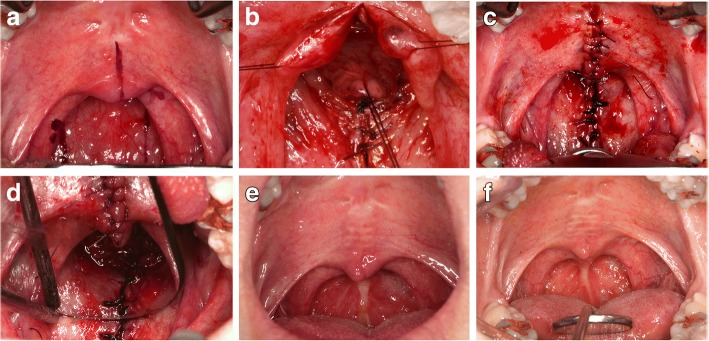
Surgical procedure of superiorly based posterior pharyngeal flap and postoperative cicatrization (case 1). **a**–**d** Surgical procedure of depicting a typical posterior pharyngeal flap. **e**, **f** Postoperative photos while phonating /a/ at 3 months and 6 months post-pharyngoplasty. At 6 months, enhanced posterior movement of the soft palate was found

**Fig. 2:**
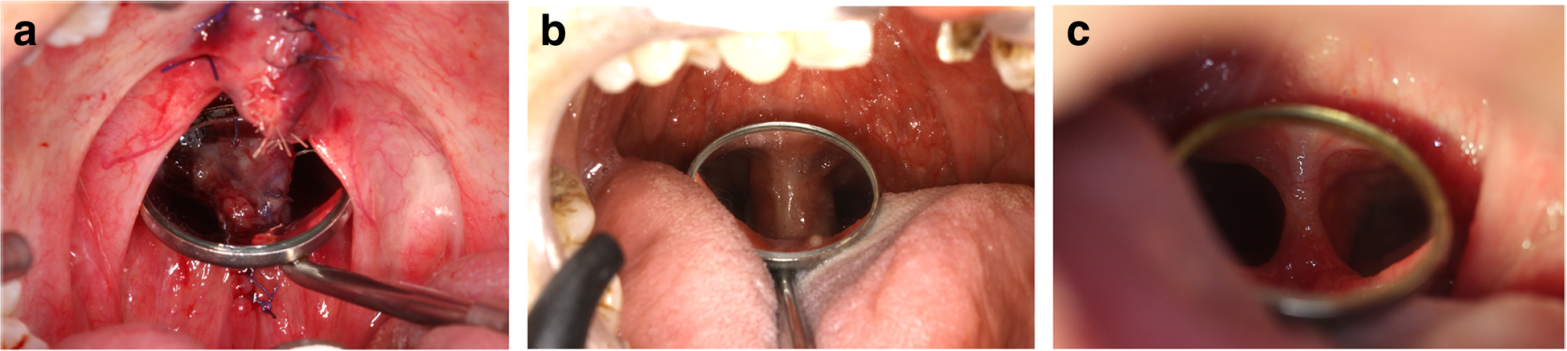
Dimensional change of the pedicle of the posterior pharyngeal flap (case 2). **a** The pedicle reflected on the dental mirror during the operation. **b** Three months after the pharyngoplasty. **c** Six months after the surgery

Both patients underwent pharyngoplasty with superiorly based posterior pharyngeal flap. Briefly, a midline incision is made in the soft palate to expose the nasal side of palatal mucosa. The width of the flap is generally 70~80% of the distance between the posterior tonsillar pillars. As suggested by Hogan, a 14 French, 4 mm catheter is inserted for lateral port size control [13]. The flap is elevated and inset to the nasal side of the soft palate using 4–0 absorbable sutures. The donor site where the prevertebral fascia is exposed is directly closed using 3–0 absorbable sutures without additional dissection of the pharyngeal wall. The incised soft palate and muscles are sutured layer by layer (Fig. 1a–d). Both patients were hospitalized for 7 days, and there were no significant events during either patient’s postoperative period. Upon return to clinic at 3 months, postoperatively, a narrowed pharyngeal flap is observed, with obvious scar tissue formation and contraction (Fig. 1e–f, Fig. 2).

### Speech and resonance evaluation

Both patients underwent speech and resonance assessment by an expert speech-language therapist, including both instrumental and perceptual evaluations. All assessments were carried out 2 weeks preoperatively, and at 1 week, 1 month, 3 months, 6 months, and 1 year postoperatively. Resonance (vowel repetitions and sentence) and articulation (consonant accuracy) were assessed.

#### Resonance test

**Fig. 3:**
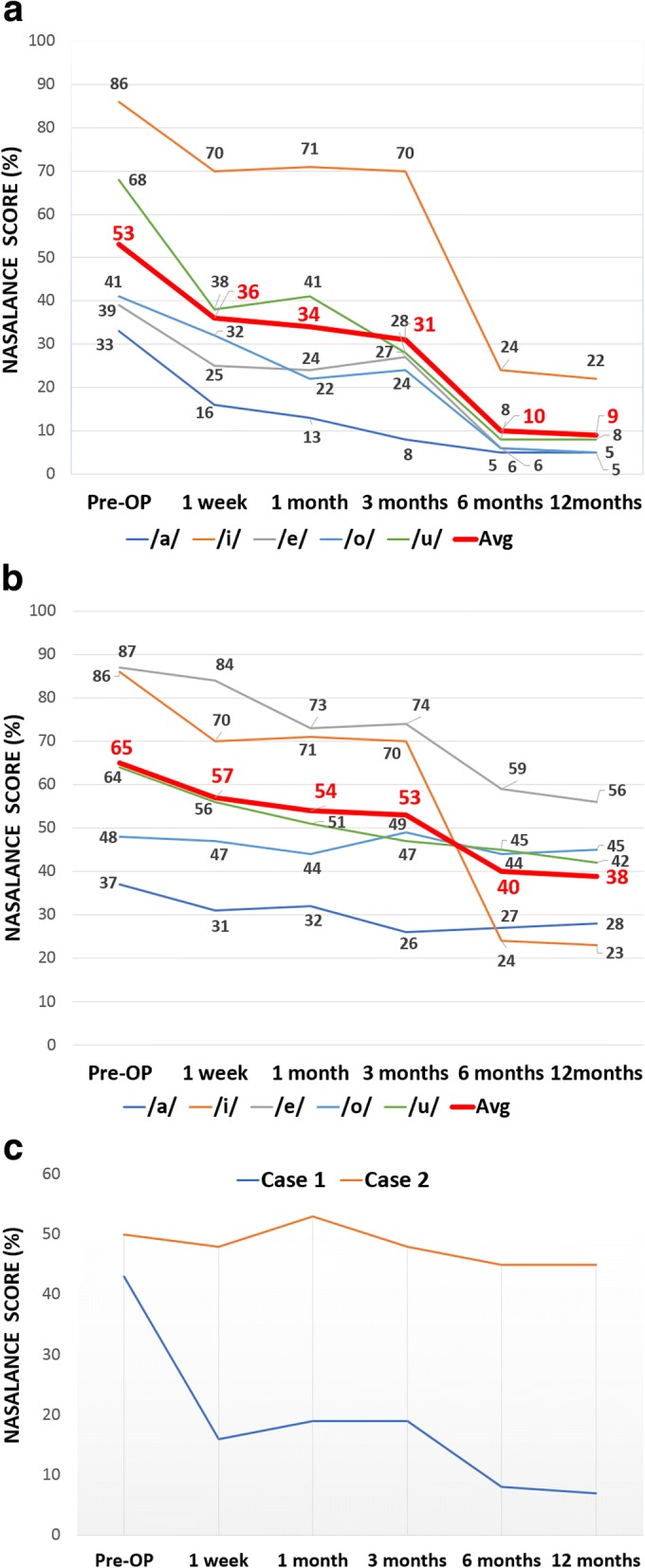
Graph of nasalance score over time. **a** Nasalance score (%) for five simple vowels and the average value (thick red line) in case 1. **b** Nasalance score for simple vowels in case 2. **c** Nasalance score measured during sentence repetition

A nasometer (Nasometer II model 6200-3, Kay Elementrics Corp., USA) was used for speech resonance testing. This device provides objective information in the form of a percentage of the total acoustic energy that is transmitted through the nose and mouth during oral speech productions. The calculated result is called the nasalance score which is the ratio of nasal acoustic energy over the combined nasal and oral acoustic energy. Nasalance scores on vowel repetitions for /a/, /i/, /e/, /o/, and /u/ and a Korean passage (/wɔljoil ohu patatkae kasɔ ʧpkɛ sɛulɯl ʧapko hwajoil sɛpjɔke tolaoketta/) were measured. Case 1 exhibited a marked decline in his nasalance score at 1 week and 6 months postoperatively. Case 2 also showed a reduction in his nasalance score, although his overall speech quality remained non-satisfactory. In addition, case 2 exhibited a reduced nasalance score for the vowel /i/ between 3 and 3 months postoperatively (Fig. 3).

#### Articulation assessment

**Fig. 4:**
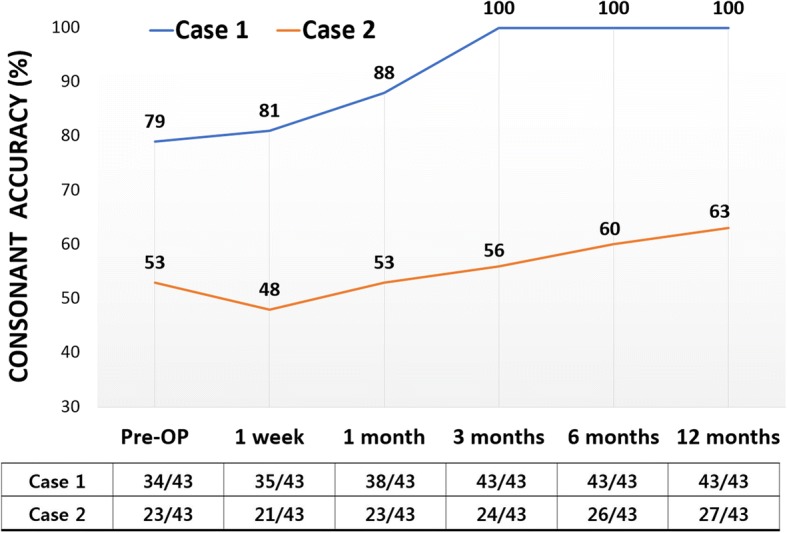
Graph of development on consonant accuracy over time. The assessment was carried out for 43 consonant sounds, and the number of correctly produced consonants was counted to calculate the accuracy (%) as presented in the table below the graph

Perceptual consonant articulation assessment was carried out by an experienced speech-language therapist. Using the document, as represented in Table 1, the therapist determined each patient’s speech error pattern by having the patients repeat words that contained the 43 Korean consonant sounds. Articulation accuracy was determined to be the number of correctly spoken consonant sounds (numerator) over 43 (denominator). Most error patterns involved distortions secondary to nasalization, with the exception of case 2 who also exhibited a substitution of /s’/ to /t’/ (Table 1). For case 1, consonant accuracy 100% after 3 months, indicating a 21% improvement. For case 2, consonant accuracy increased by 10% which are four consonant sounds (Fig. 4).

**Table 1:** Example table for consonant accuracy evaluation

Consonant	Accuracy: 53% (23/43)																			
Error pattern S: substitution D: distortion O: omission A: addition		p/b	pp	p’	m	n	H	k/g	kk	k’	t/d	tt	t’	ng	s	ss	Ch/j	tch	Ch’	l/r
	Initial sound	D	D	D				D	D	D	D		D		S	D		D	D	
	Middle sound		D						D			D	D		D	D	D	D		
	Final sound

## Discussion

In spite of advances in primary palatoplasty and speech-language therapy, hypernasality may remain, following surgery for VPI. At this point, prosthetics such as speech aids can be considered to enhance VP function. In cases of mild VPI, proper use of prosthetics, along with speech therapy, can produce satisfactory results. However, as Shin suggested, patients with severe hypernasality (indicated by nasalance scores of 60% or more) often require surgical interventions [5, 14, 15]. Cases 1 and 2 exhibited preoperative nasalance scores of 53 and 65%, respectively. According to Shin’s criteria, case 1 could consider the use of prosthetics. However, he desired a more rapid outcome and therefore was referred for surgery (Table 2). Of the available surgical techniques, the superiorly based posterior pharyngeal flap is the most common. This method is more effective when patients have insufficient anterior-posterior pharyngeal wall movement, with relatively good movement of the lateral pharyngeal walls [6, 16]. Since both cases had poor lateral movement, the posterior pharyngeal flap was selected.

**Table 2:** Degree of nasality and recommended intervention methods (Shin’s criteria)

Nasalance score (%)	Degree of nasality	Recommended treatment
~ 20	None	No intervention
20~35	Mild	Speech therapy
35~45	Moderate	Speech aid appliance with speech therapy
45~60	High	Surgery or speech aid
60~	Severe	Surgery

As presented in this report, nasalance scores steadily declined until 1 year postoperatively. The average nasalance curve for five vowels increased by the first week and between 3 to 6 months postoperatively. The graph of nasalance scores during sentence repetition does not clearly reflect this pattern for case 2 since he experienced dramatic reductions in nasalance for the vowel /i/; however, the sentence stimuli contained only a few instances of /i/. The first-week changes seemed reasonable because the original flap size was maintained during this period, while reducing the VP port size. On the other hand, the second decline in nasalance after 3 months was not as easy to explain considering the obvious narrowing of pharyngeal flap pedicle by scar contracture. Past report indicated 49% width reduction in cases of the superiorly based pharyngeal flaps [17]. Furthermore, the flap can eventually assume a tube shape because of scar contractures, tethering the soft palate to the posterior wall in an unfavorable inferior direction [17]. On the other hand, a report revealed that scar contraction of the pharyngeal flap can aggravate nocturnal obstructions because pharyngeal flap contraction can lengthen the soft palate [18]. Even given the unsatisfying reduction in the width, contracture of the flap bridge may facilitate posterior movement, and scar tissue formation on the posterior wall could form a hump that reduced the size of the VP port, as seen in the case 1 figure (Fig. 1e–f). The combination of dimensional change of the VP structure with improved lateral wall movement may account for the continuous enhancement of hypernasality found in this report. However, currently, there is no objective evidence to support the hypothesis that the scar formation can decrease the dimensions of the VP; therefore, additional research should be considered.

The cases also demonstrate improvement in consonant accuracy since distortion by nasalization was the main cause of the patients’ articulation disorders. The consonant accuracy test perceptually evaluates each consonant sound to be either correct or false. Therefore, gradual change or slight improvement in nasality does not directly reflect consonant accuracy. In case 1, consonant articulation normalized when the average nasalance score went down to the 30%. However, follow-up studies in large groups are necessary to support this finding.

Multiple factors can explain why case 1 showed a better result than case 2. The first subject had an incomplete cleft palate which is a less severe deformity than the complete cleft of case 2. Also, case 1 had a normal dental arch without missing teeth. Furthermore, though it might have been for a short period, 6 months of speech-language therapy was another positive factor. Thus, a preoperative test of consonant accuracy revealed a 26% difference between the cases, compared with a 10% difference compared to the preoperative nasalance scores. Postoperative speech improvement was much greater in case 2 as well. The delayed primary cleft repair at around the age of three and pharyngoplasty at age 49 were also another unfavorable factors for case 2. However, the severe hypernasality of case 2 improved to a moderate level of hypernasality which will hopefully continue to improve through active speech therapy with the prosthetic device [5].

## Conclusions

Generally, when managing patients with VPI who have both resonance and articulation disorders, treatment for the resonance disorder usually precedes articulation training. However, if surgical intervention is not immediately necessary, appropriate and active speech-language therapy after primary repair of the cleft palate is strongly recommended. Even though scar contracture may reduce the width of the flap over time, superiorly based posterior pharyngeal flaps are good surgical options for patients with severe hypernasality. Since, as shown in this report, the postoperative declines in nasalance scores can last at least 6 months, longtime follow-up with speech training should be recommended to patients.

## Abbreviations

VP function: Velopharyngeal function
VP port: Velopharyngeal port
VPI: Velopharyngeal incompetence; velopharyngeal insufficiency
